# Concurrent Dengue and Malaria

**DOI:** 10.3201/eid1107.041352

**Published:** 2005-07

**Authors:** Remi N. Charrel, Philippe Brouqui, Cedric Foucault, Xavier de Lamballerie

**Affiliations:** * Université de la Méditerranée, Marseille, France;; † AP-HM Hôpital Nord, Marseille, France

**Keywords:** dengue, malaria, concurrent infection

**To the Editor:** A 37-year-old woman, a logistics director for a nongovernment organization, returned to France in March 2004 from an 18-day trip to Guinea, Senegal, and Sierra Leone. Fever, chills, and myalgia developed in the woman 3 days before she returned to France, and she treated herself with aspirin and paracetamol (acetaminophen). Malaria prophylaxis was taken neither during nor after the trip.

The day after returning to France, the woman's condition progressively worsened; diarrhea and extreme weakness that led to the inability to walk developed. Ten days after her return, she was admitted to the local hospital and treated with intravenous quinine and oral doxycycline (2 g per day) after thick and thin blood films showed 3% parasitemia with *Plasmodium falciparum*. Three days later, she was still febrile and had conjunctival jaundice, vomiting, insomnia, and moderate hemorrhagic manifestations (epistaxis, blood in urine and feces). Three days after initial hospitalization, the patient was transferred to the Infectious Diseases Unit in Marseille; fever (39.5°C) continued, and hepatosplenomegaly developed. Biologic analyses showed disseminated intravascular coagulation with platelet count of 22,000/mL, an elevated prothrombin time (54% higher than the control value), a longer activated clotting time (51 seconds versus a control value of 34 seconds), a fibrinogen level of 0.9 g/dL, exaggerated plasma fibrin formation and degradation, and hepatic cytolysis with both aspartate aminotransferase and alanine aminotransferase levels of 80 U/L.

Although acute malaria had been diagnosed, viral serologic tests were performed because the patient had returned from a tropical country with a fever. Persons in these circumstances are systematically administered a series of tests to determine the cause of their fever. Serologic tests for dengue performed on the acute-phase serum (collected 13 days after onset of symptoms) and convalescent-phase serum (collected 23 days after onset of symptoms) showed the presence of immunoglobulin (Ig) M (titers 1:800 and 1:3,200, respectively) and IgG (titers 1:400 and 1:3,200, respectively), which suggested that the patient had dengue fever and malaria concurrently. These results were obtained by using the Dengue Duo IgM-capture and IgG-indirect enzyme-linked immunosorbent assay (Biotrin, PanBio Pty. Ltd., Brisbane, Australia). The same acute-phase serum was tested for flavivirus RNA by seminested reverse transcription–polymerase chain reaction (RT-PCR) by using flavivirus consensus primers PF1S and PF2R as previously described (1) in conjunction with the sense primer PF3S (GCIATHTGGTAYATGTGGYT). Attempts to isolate viruses by using C6/36 and Vero cells were unsuccessful, which might be expected given the delay between the onset of symptoms and specimen collection.

Sequence analysis of the 163-bp (primers excluded) PCR product (GenBank accession no. AY862501) showed 89%–99.4% range of homology with 34 dengue 3 virus strains by using the BLAST nucleotide program. Similarities obtained with sequences of dengue virus 1, 2, and 4 were ≤87%. Phylogenetic analysis performed with the patient sequence together with homologous sequences from dengue viruses and other flaviviruses showed that it corresponded to dengue 3 virus species. RT-PCR amplification on the convalescent-phase serum was negative. Based on World Health Organization criteria, the patient was diagnosed with dengue fever (2). The patient's interview showed a previous dengue fever episode in Haiti in 1995 and a previous malaria episode in Burundi in 2002, but biologic confirmation was not available, and serum was not collected before this episode. Therefore, we could not determine definitively whether this patient experienced primary or secondary dengue. In light of virologic tests results, the diagnosis of secondary dengue infection was more likely (3).

A PubMed search using the keywords dengue, mixed infections, dual infections, simultaneous infections, and concurrent infections retrieved 14 references published since 1958. In most cases, concurrent infection was with 2 dengue virus strains from 2 different serotypes in a single patient (4,5). Only 6 published studies reported concurrent infection with dengue virus and a bacterium (*Salmonella typhi*, *Shigella sonnei*, *Leptospira* spp.) (6–8) or with a virus such as Chikungunya virus (9).

To our knowledge, this is the first report of mixed dengue–parasite infection, dengue virus with *P. falciparum*. The authors previously questioned the accuracy of a serologic test to diagnosis dengue fever in patients experiencing malaria because reactivity was nonspecific on certain rapid serologic assays (10); however, serologic tests used in this study have demonstrated good specificity (10), and molecular tests are not prone to such specificity problems. Classifying this case as dengue hemorrhagic fever is questionable since some of the hemorrhagic signs may have been caused by acute malaria. In cases of concurrent infections involving a dengue virus, questions related to the influence of mixed infection on severity and prognosis are, therefore, impossible to address because of lack of information. Further investigations are required because this situation likely occurs frequently in nature, despite scant available data.
